# Relational Therapy in Post‐Incarceration Reentry: A Scoping Review

**DOI:** 10.1111/jmft.70167

**Published:** 2026-08-21

**Authors:** Eman Tadros, Thita Lamsam, Chandni Anandha Krishnan

**Affiliations:** ^1^ Syracuse University Syracuse New York USA; ^2^ Northwestern University Evanston Illinois USA; ^3^ University of Wisconsin Madison Madison Wisconsin USA

**Keywords:** marriage and family therapy, post‐incarceration, scoping review

## Abstract

An estimated 600,000 individuals returning home after incarceration each year face significant challenges that constrain their reentry and reintegration into society. A scoping review was conducted on how relational therapy is discussed in the literature on post‐incarceration. Utilizing the Preferred Reporting Items for Systematic Reviews and Meta‐Analysis, 19 out of 304 articles met the inclusion criteria for full review. Our findings highlight the limited empirical evidence on relational therapy interventions for post‐incarceration. The review also emphasizes that there is a lack of evidence‐based therapeutic models or interventions that marriage and family therapists can utilize when working with this population. The paper sheds light on this issue and calls for more research on relational therapy geared towards those post‐ carceral release in the United States.

## 1 Introduction

Although the U.S. accounts for only 5% of the worldwide population, it holds 25% of the world's incarcerated population (Kirk and Wakefield 2018). Each year, approximately 600,000 individuals are released from prison, many of whom seek to reunite with their families (Carson and Golinelli 2013). However, this reunification process is often complicated by internal and external stressors. Formerly incarcerated individuals commonly face stigma surrounding their criminal record (Tadros et al. 2025) and pressures from partners to contribute financially immediately upon release (Cooper‐Sadlo et al. 2019; Yocum and Nath 2011). These challenges can intensify existing mental health vulnerabilities and lead to decreased relationship satisfaction, higher rates of domestic conflict, and increased divorce (Few‐Demo and Arditti 2014).

## 2 The Role of Relational Family Therapy

Systemic or relational family therapy focuses on the quality of interpersonal relationships as a central component of psychological health (Varghese et al. 2020). Unlike traditional individual‐focused therapy models that prioritize individual autonomy, relational therapy emphasizes mutuality, empathy, and connection as foundations for emotional regulation and resilience (Hammer 2012). These relational skills are essential for managing stress and preventing mental health challenges like depression and anxiety. Relational therapy is especially crucial for families impacted by incarceration (Negash et al. 2026; Tadros et al. 2019; Umamaheswar et al. 2025).

Challenges such as unmet expectations, communication breakdowns, and emotional distance often stem from a lack of mutual understanding within the family system. Family therapy has been shown to restore fractured parent‐child relationships, improve youth behavior, and reduce symptoms of substance‐use disorders (Wesselmann et al. 2018). Despite its benefits, access to family therapy for formerly incarcerated individuals remains limited. Incarceration, while often framed as rehabilitative, frequently disrupts family bonds and imposes psychological trauma on children and partners (Liu et al. 2026). Without adequate support, reintegration into the family can be overwhelming, particularly in the absence of therapeutic interventions (Umamaheswar et al. 2025). Expanding and destigmatizing access to relational family therapy is essential for supporting healthy reunification and promoting long‐term family stability (Tadros 2024).

### 2.1 Barriers and the Need for Support

Studies reveal that men face limited income opportunities during incarceration and struggle to secure employment and fair wages post‐release (Beckett and Goldberg 2022; Massoglia and Remster 2019). These issues lead to financial difficulties and reduced self‐worth due to the inability to support their families (Turney 2015). Partners of incarcerated men bear extra responsibilities, resulting in financial strain and relationship stress (Bruns and Lee 2020; Tadros and Presley 2024). Women with incarcerated partners are more likely to work multiple jobs than women in otherwise similar circumstances (Bruns 2019). These stressors further increase the challenges that individuals face post‐incarceration. Supportive communication skills are associated with relationships becoming stronger and happier, at least from the perspective of nonincarcerated heterosexual women experiencing intimate partner incarceration, and healthy intimate relationships among the incarcerated population may be a mechanism of resilience with the potential to promote healthy post‐carceral adaptation (Durante et al. 2023).

Children of incarcerated parents are especially impacted by these disruptions. The reentry process requires an adjustment to a new or altered parental role, which can negatively affect a child's emotional and social development. These children are more susceptible to behavioral issues, social isolation, and lower academic performance (Cochran et al. 2018). Furthermore, the parents' ability to provide a stable and emotionally available presence is critical in reducing the intergenerational cycle of incarceration. Given that Black children are nine times and Hispanic children three times more likely to experience parental incarceration compared to White children, these dynamics disproportionately harm racial and ethnic minority families and contribute to widening socioeconomic disparities (Warren et al. 2019).

The lack of accessible relational therapy exacerbates these struggles, leaving families without the tools needed to rebuild trust and connection. Transitional programs that incorporate mental health care and substance use treatment have proven effective, reducing recidivism rates for women to 17%, compared to 50% without such programs (Edwards et al. 2022; Mejia et al. 2024). Participants in these programs also reported sustained feelings of social connectedness 6 months post‐release (Mejia et al. 2024), displaying the critical role of relational support in successful reintegration. Therefore, relational therapy is significantly underutilized as an intervention for supporting formerly incarcerated individuals and their loved ones.

## 3 The Current Study

Currently, there is a lack of literature that explores the impacts of incarceration on individuals after release, specifically relational therapy (Greenwood 2016). This is of concern because incarceration can have a long‐lasting negative impact on the incarcerated individual and their support system, even years after release. There is a lack of supportive care, specifically relational therapy programs for individuals who have recently been released from a correctional facility (Tadros et al. 2021). In addition, marriage and family therapists (MFTs) are often not provided training and thus are not well‐informed about the lingering effects of incarceration and the impact it has on relationship development post‐release (Negash et al. 2026; Tadros and Owens 2021), making it difficult to develop effective interventions and improve overall mental health and well‐being. Additionally, with the staggering number of individuals who are released from prison each year, MFTs are likely to come across loved ones of incarcerated individuals. Systemic interventions geared towards the transition between correctional facilities and the community, while addressing larger preexisting relational issues, are essential to avoid recidivism, indicating a successful reentry process. This scoping review will explore how relational therapy is discussed in the literature on post‐incarceration. Therefore, we seek to answer how relational therapy is discussed in the literature on post‐incarceration.

## 4 Methods

The literature search conducted utilized the Preferred Reporting Items for Systematic Reviews and Meta‐Analyses (PRISMA) procedures to model the scoping review process (Mak and Thomas 2022; Munn et al. 2018). After careful thought and consideration of the weight between the decision to do a scoping or systematic review, we decided on a scoping review versus a systematic review because of the scarcity of explicitly relational or family therapy research with this population. In addition, the methodological choice to map the overview of relationally relevant post‐incarceration literature, rather than synthesize a defined clinical intervention, is consistent with a systematically conducted scoping approach. Thus, this rationale is much more consistent with established guidance on scoping reviews (Munn et al. 2018), which are particularly appropriate when the goal is to identify the types of available evidence, clarify concepts, and map gaps in emerging or underdeveloped areas.

Following similar topical literature, an electronic literature search was conducted using the database PsycINFO, which followed the procedure of other articles that used a similar systematic methodology as well as discussed the topics related to family science (Kaur et al. 2019; Tadros et al. 2023; Tadros and Ogun 2026). In July 2024, we compiled studies that fit the inclusion search criteria that were published between 2014 and 2024. Studies were limited to peer‐reviewed academic articles (no books, magazines, or dissertations) and had to be in the English language. Search terms utilized were: Interventions OR techniques OR therapy, and post‐incarceration OR post‐incarceration OR post‐release OR post‐release OR formerly incarcerated. While our initial search terms included relational and systemic‐related keywords, our preliminary search result yielded four articles within the PsychINFO database, which confirmed the anticipated scarcity of explicitly relational studies in this field, necessitating an assessment of relational and systemic relevance during the abstract and full‐text review stages. Because of this, relational and systemic terms were purposefully omitted from the search string to prevent the over‐restriction of results. The revised search yielded a total of 304 articles. Typically, the next step would be the removal of duplicates; however, none appeared.

An abstract review was conducted by all authors to identify articles that coincided with the inclusion criteria: must discuss the post‐incarceration population and/or its direct/indirect system, utilize a relational/systemic theoretical model/lens, and/or discuss a relational therapeutic intervention/techniques, and/or address the impacts of incarceration on mental health and psychological well‐being after release. Given the breadth of these criteria, it is important to note that this range was intentional because evidence‐based relational therapy for this population is markedly underrepresented in the literature. A broader inclusion framework was necessary to accurately map what exists, document where the gap lives, and identify the relational threads embedded in adjacent scholarship. Additionally, studies involving interventions initiated during incarceration were included when their stated purpose was to facilitate post‐release reentry outcomes, as the reentry process is understood to begin prior to release.

**Figure 1:**
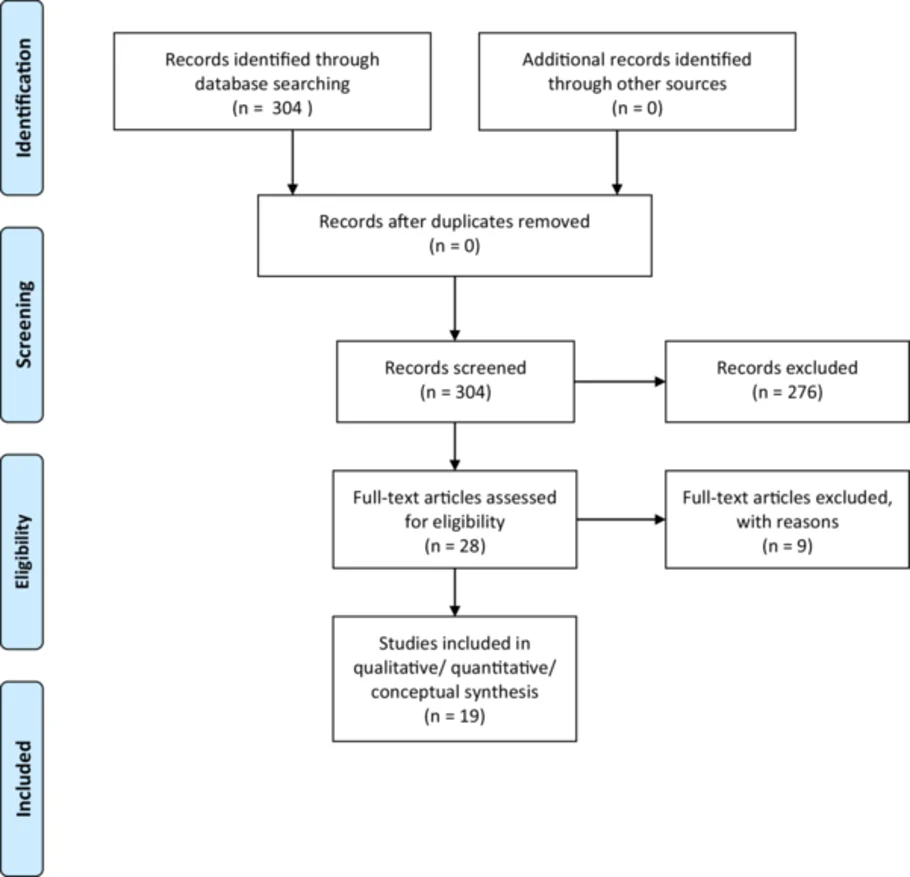
PRISMA flow chart. [Color figure can be viewed at wileyonlinelibrary.com]

As a result, a total of 28 articles qualified for a full‐text review. After reading the full texts and assessing for compliance with the inclusion criteria, 9 articles were excluded, resulting in 19 articles that met the criteria. Studies included qualitative, quantitative, and conceptual/theoretical articles. Three researchers assessed for inclusion criteria independently and then reviewed and compared their findings to ensure credibility in the analysis. Research team meetings were frequent and followed best practices, as scholars have recommended that researchers are encouraged to practice reflexivity throughout the research process to ensure alignment with the principles of scoping reviews (Mak and Thomas 2022). Figure 1 summarizes the search process (Moher et al. 2009).

## 5 Results

The following paragraphs summarizes the articles that explore the post‐incarceration population through a relational lens. It is important to highlight that relational therapy, or systemic interventions were not the emphasis of the majority of the articles. While some of the articles proposed relational interventions, many of them did not. All three researchers reviewed the 19 articles that met the inclusion criteria, regardless of the amount of attention on the topic of interest, to allow for an accurate depiction of what is in the available literature. These articles are organized according to the stage of the incarceration–reentry process (preparation for reentry, transitional, and reintegrative) represented in the population being examined. An additional section (contextual) includes studies that examine the relational systems surrounding incarcerated individuals (e.g., children, families, clinicians) who are impacted by incarceration despite not being incarcerated themselves. Further, the discussed articles emphasize the same gap of relational therapy in reentry and evidence‐based therapeutic modalities.

### 5.1 Preparation for Reentry

The preparation for reentry section explores interventions delivered to incarcerated individuals during their time in a correctional setting. While this is not necessarily considered post‐incarceration, these preparatory interventions are essential in assisting an individual in a successful reentry. Brubacher et al. (2023) conducted a theoretical study on the importance of communication skill development among incarcerated mothers, specifically the verbal aspect of communication. The study discussed how improved communication can facilitate many of the factors associated with desistance, such as life stability and the creation of redemption narratives, which correlated with theories of desistance on an individual/agentic and social/structural level. Researchers suggested interventions based on ecological systems theory that utilizes a whole‐systems approach with a trauma‐informed lens, which allows one to look beyond surface needs and predict the utility of the intervention in multiple contexts. These interventions can have wide‐ranging benefits to the mothers, their children, and other close relationships, lowering the likelihood of recidivism.

Armstrong et al. (2018) conducted a systematic review and meta‐analysis examining the effectiveness of parenting programs implemented during incarceration. The systematic review synthesised research on interventions designed to help incarcerated parents maintain and strengthen relationships with their children while in custody, while the meta‐analysis quantitatively assessed the impact of these programs across studies. Overall, findings suggested that parenting programs delivered in correctional settings were associated with improvements in parenting knowledge, parenting attitudes, and the quality of parent–child relationships. Some evidence also indicated that these programs supported parents' confidence in their parenting roles and increased their motivation to remain involved in their children's lives following release. Because these interventions occur during incarceration and are intended to prepare parents for reunification with their families, the authors highlighted the importance of incorporating structured parenting programs into correctional systems as a preparatory step for successful family reintegration after release.

When considered collectively, the studies in this section have a similar theoretical focus on systems‐level and ecological thinking. Both Brubacher et al. (2023) and Armstrong et al. (2018) place the individual within a larger relational context that encompasses family, community, and institutional systems, whether the individual is a parent fortifying ties with a child or a mother honing her own communication skills. Most importantly, both interventions are situated within correctional settings, which influences what is therapeutically feasible. Because of the structural limitations of the carceral environment, goals at this stage are typically preparatory and skills‐focused rather than deeply relational. The therapeutic relationship itself must be negotiated in an institutional setting that does not place a high priority on relational healing, and access to children and family members is restricted. However, these studies show that even within such constraints, relational and systemic interventions can yield meaningful improvements in parenting knowledge, communication, and family motivation. The groundwork for relational repair can begin before release, even if its fuller expression must wait until after. Furthermore, neither study examined the long‐term relational outcomes of these in‐custody interventions, pointing to a gap in understanding how preparatory work inside carceral settings connects to post‐release family functioning.

### 5.2 Transitional

This section highlights studies that explore programs and factors that are systemically beneficial to incarcerated individuals prior to and immediately after release. Menting et al. (2024) conducted a longitudinal study to evaluate the long‐term preventative effectiveness of the Better Start program, a parenting intervention in the Netherlands for mothers of 2‐ to 10‐year‐olds during the period when mothers leave incarceration. The intervention, based on social learning theory, aims to prevent disruptive behaviors, including delinquency in children, as well as recidivism in mothers. Results found that children of mothers in the Better Start program showed less delinquent behavior in adolescence compared to children of mothers who did not receive the intervention. Findings suggest that an evidence‐based parenting intervention, when adjusted to suit the population, can have long‐term positive impacts on families affected by maternal incarceration.

Pettus‐Davis (2021) proposed Support4Families, a systemic intervention model designed to support members of the family in the reentry process. Support4Families aims to allow family members to learn, practice, and integrate skills and tools into their lives prior to the loved ones' release, later refining the tool application skills with professional help after release. This intervention is guided by the Main Effect Model of Social Support, which proposes that social support is the product of an active integration into social networks (e.g., families), along with the Family Resilience Framework, which informs the mechanisms by which the family unit can develop resilient and adaptive support strategies. The article highlighted utilizing a family systems perspective, as reentry does not occur in isolation. The program was stated to be undergoing pilot testing, aiming to create family stability and individual and family well‐being through providing knowledge, tools, skills, and resources that family members will need.

Maschi and Koskinen (2015) conducted a qualitative study to explore factors that influence successful community reunification of older people who were released from prison. Participants reported that internal and external resources or support influenced the likelihood of successful reunification with their families, peers, and communities and their ability to manage obstacles. Results aligned with social support theory, which highlights the importance of external resources and supports. Findings suggested that programs for aging people, implemented both in prison and after release, will be more beneficial if they incorporate the perspectives and lived experiences of formerly incarcerated older people into the program design. The study highlighted how professionals within aging, health, and criminal justice services can play a central role in prevention, assessment, and intervention to reunite incarcerated aging people with their families and communities.

Wilson et al. (2024) piloted the Passport to Freedom (P2F) program, a woman‐centered, trauma‐informed reentry initiative guided by the Eco‐Social Trauma Intervention Model. Using a two‐phase mixed methods design, they developed and tested the program. Results showed reduced depression and everyday stress among participants. Findings highlight the need for evidence‐based, trauma‐informed support to help incarcerated women address mental health challenges and reduce recidivism risk upon reentry.

Wallace et al. (2016) explored how family support during and after incarceration is related to post‐release mental health. The study examined the relationship between social support and personal health through the main effects model and the stress‐buffering model. Results implied that while familial social support during incarceration had no association with mental health post‐release, post‐release familial support did impact post‐release mental health, with negative familial support being more consequential than positive familial support. The study also found that the change in support during and after incarceration had an effect on mental health. Further, for better mental health outcomes post‐release, families should enhance positive ties while simultaneously lowering negative support. Programs that help families and formerly incarcerated individuals cope with the strains of reentry are likely to improve familial relationships and promote better mental health outcomes for reentering civilians.

The studies in this section collectively emphasize that the transitional period, which encompasses the time immediately preceding and following release, is an essential and distinctly intricate phase for relational intervention. Some of the programs investigated here, like Support4Families (Pettus‐Davis 2021) and the Better Start program (Menting et al. 2024), purposefully bridge the line between incarceration and community reentry by involving both the person incarcerated and their family system across the release threshold. An important conceptual insight is reflected in this dual‐phase design, which is that reentry does not start at the moment of release but rather is a process that must be cultivated before it takes place.

The studies additionally demonstrate that the transitional setting is ambiguous; interventions are not completely inside or outside of carceral control, and this ambiguity affects both engagement and therapeutic framing. Maschi and Koskinen (2015), for instance, advocate for programming that spans both prison and post‐release contexts for aging individuals, recognizing that continuity of support is more effective than discrete, stage‐specific services. Wallace et al. (2016) caution against oversimplifying family support as inherently beneficial, demonstrating that its quality, not merely its presence, determines its impact on post‐release mental health, with negative familial support proving more consequential than positive support. The combination of these findings indicates that relational interventions during the transitional phase must not only address skill‐building but also the quality and history of family relationships, including their potential for both healing and harm. Despite the relational complexity these studies reveal, none utilized a specific family therapy modality, highlighting the broader gap this review aims to document.

### 5.3 Reintegrative

This section discusses interventions for incarcerated individuals that begin after release from incarceration, including those on parole/probation. Tadros et al. (2022) conducted a clinical case study surrounding a formerly incarcerated client displaying symptoms of specific phobia. They utilized narrative therapy, focusing on deconstructing and reauthoring personal stories, as the method of intervention. Collaboratively, the therapist and client worked on understanding how family‐of‐origin issues played a role in formulating his self‐narrative, which utilized his phobia to project anxiety on. The study found narrative therapy to be highly effective in helping the client reframe his narrative, leading to a reduction of specific phobia symptoms and anxiety. Findings suggested a need to break barriers to access to mental health services after release from incarceration, along with utilizing a more systemic lens in a therapy setting.

Crain et al. (2024) explored the self‐reported experiences of formerly incarcerated men of color working as personal fitness trainers to White clientele. This study utilized Critical Race Theory, which draws attention to the over‐policing of communities of color and its contribution to the mass incarceration of men of color, reinforcing stereotypes, evoking racial threat, and encouraging endorsement of more aggressive or biased policing practices. All 10 participants reported experiencing microaggressions in the workplace or community. The study emphasized the challenges faced by men of color returning to their communities and emphasized the benefits of environments where formerly incarcerated individuals can develop resiliency skills to disrupt the cycles of recidivism.

Welch et al. (2019) conducted a qualitative study and interviewed participants on their lived parental experiences as a formerly incarcerated Black father. Results found three overarching themes in the discussion: (1) past experience influenced parenting in the present; (2) contact with children was consistent; and (3) personal growth contributed to parental growth. Findings called for more strength‐based parenting programs within transitional living and residential programs that support members of the reentry population. Parenting classes that examine fathers' experiences in their family of origin may be one way to explore intergenerational patterns passed down.

Gurusami (2019) conducted an observational study with formerly incarcerated women to examine how mothers experience discursive forces in daily post‐incarceration lives. The author drew from theories of “motherwork” and explored three different types of decarceral motherwork during the observation period: collective, hypervigilant, and crisis. Findings suggest that formerly incarcerated Black women face obstacles after incarceration that complicate mothering, with the biggest factor shaping their parenting approach being their need to prove to state agencies that they were fit to have custody of their children. As a result, mothers often drew from one of the three types of decarceral motherwork (collective, hypervigilant, and crisis). This study demonstrated the constraints of labor under the carceral state and revealed the broader context of social problems produced from state surveillance and how individuals and families navigate their survival within this context.

Meza et al. (2023) conducted a mixed‐methods study to examine the relationship between perceived neighborhood violence and health among African American and Latinx youth undergoing reentry. This study utilizes Bronfenbrenner's ecological systems theory by exploring the relationship between the microsystem (perceived neighborhood violence) and the individual (health). Quantitative analyses found that more than half of the youth undergoing reentry reported perceived exposure to neighborhood violence, which was linked to a wide range of physical health and psychosocial well‐being. They also found that perceived neighborhood violence was associated with poor psychosocial well‐being, including more perceived stress and less family support, which can be partially explained by increased time spent at home, leading to more opportunities for family conflict. Findings show that understanding barriers to treatment, as well as introducing family telehealth interventions, can improve both substance use outcomes and family cohesion.

Cooper‐Sadlo et al. (2019) conducted an in‐depth interview with 12 mothers to understand their experiences of substance use, incarceration, and reunification with their children. Using the Family Resilience Framework, which provides a lens to understand the importance of using a strength‐based approach when considering the sociocultural context in which families function through supporting individuals and families in making meaning from their adverse experiences. Results found that all of the participants reported love and a desire to protect, care, and provide for their children, experiencing guilt and shame when not fulfilling mothering duties, and the importance of acceptance and forgiveness in rebuilding and restructuring their families. Findings highlight the need for family therapists to continue applying a broad, integrative view of systems to formerly incarcerated mothers with substance use disorder, allowing for interventions at multiple systems of care. They emphasized the need to address these mothers' relational and emotional needs of their family members at equal importance as the mothers' survival needs.

Salem et al. (2019) conducted a cross‐sectional study to explore physical, psychological, and social frailty among formerly incarcerated, currently homeless women exiting California jails and prisons, who have a history of drug use and addiction. This study explored the Frailty Framework among Vulnerable Populations, which explains how individual‐level, situational, behavioral, health‐related, biological, environmental, and resource‐related factors influence adverse outcomes of physical, psychological, and social domains. The findings shed light on how physical, psychological, and social frailty are significant issues among formerly incarcerated, currently homeless women. The study emphasized how trauma‐informed and specific care, along with case management tailored to help frail women learn self‐care strategies during reentry, can be beneficial in the long run.

Pettus‐Davis et al. (2017) examined the patterns of family and nonfamily social support among incarcerated versus nonincarcerated emerging adults. The study used Cullen's (1994) social support theory, which suggests that positive relationships are a key factor for formerly incarcerated emerging adults to navigate away from criminal behaviors during reentry. Results show that justice‐involved emerging adults demonstrate much higher support from family than from nonfamily adults. The study also found that formerly incarcerated emerging adults experience greater declines in nonfamily social support than their nonincarcerated counterparts, and longer incarceration stays were associated with greater social support deterioration. The study highlights how those with incarceration histories are less likely to receive social support, and how interventions should help formerly incarcerated clients match their support needs with the resources they have in their social network.

Valera and Boyas (2019) explored what types of social support factors (perceived social support, community support, and religious support) significantly predict mental health and well‐being outcomes through 225 formerly incarcerated men of color in New York City. Results suggest that increased social support, community support, and religious support were significantly associated with lower levels of psychological morbidity. The existence of perceived social support had the most significant impact on mental health, potentially due to how family relationships play a substantial role in helping older incarcerated men cope with adversity. Results also found that a majority of the participants did not report using mental health services despite taking medication for depression, anxiety, anger, and bipolar disorder. Findings highlight the need for increased supportive care post‐release for individuals with and without social support networks and access to mental health services.

Among the reintegrative studies, multiple convergent themes emerge that no individual article comprehensively addresses. To begin with, structural and racial barriers are widespread and interconnected. Crain et al. (2024), Gurusami (2019), Meza et al. (2022), and Welch et al. (2019) all focus on the experiences of people of color and consistently show that systemic factors like over‐policing, state surveillance, neighborhood violence, and racial microaggressions affect reintegration in ways that models that focus on individuals can't fully explain. All of these studies ask for frameworks that take into consideration the environment, the community, and race, but none of them directly tested a relational or family therapy intervention. Second, social support consistently predicts reintegrative success across studies (Pettus‐Davis et al. 2017; Valera and Boyas 2019). However, the mechanisms for fostering or restoring support following the relational disruptions of incarceration remain insufficiently theorized and inadequately addressed in practice. Third, the interventions in this section, like narrative therapy (Tadros et al. 2022), telehealth (Meza et al. 2022), and strength‐based parenting (Welch et al. 2019), are all provided in community‐based settings after release, which allows for deeper relationships and more therapeutic options than the carceral and transitional settings discussed earlier.

### 5.4 Contextual

The term “contextual” refers to the fundamental philosophical tenet of the therapy, which is that human beings cannot be fully understood in isolation (Boszormenyi‐Nagy and Krasner 1986). Instead, contextual family therapy urges families and individuals to be understood in the context of their interpersonal connections, heritage, and intergenerational ethical obligations. Therefore, the Contextual section explores interventions for support systems of the incarcerated individual (e.g., children with incarcerated parent(s), mental health providers) that have been directly or indirectly impacted by an individual's incarceration.

Nguyen et al. (2023) highlighted the multidimensional reality of parental incarceration through their cross‐sectional analysis. Using Bronfenbrenner's process‐person‐context‐time (PPCT) model, the study explored the impact of neighborhood‐based social infrastructure and social support on school engagement of children with parents or guardians who have experienced incarceration (CPGI). Descriptive analysis demonstrated that parental incarceration is not an isolated issue, as having a caregiver incarcerated may affect a child's developmental trajectory as much as other aspects of life. CPGIs are more engaged at school when their families live in a supportive neighborhood and have access to social capital, and show higher school engagement when living in neighborhoods with social infrastructures such as sidewalks. The study emphasized the importance of having a reliable social support network or individual person during times of adversity, being particularly helpful for CPGI and their families.

Hiolski et al. (2019) compared behavioral indicators of physical health in youth with currently and formerly incarcerated parents to youth with no history of parental incarceration. Findings suggest there is a significant association between parental incarceration and lower levels of healthy behaviors and higher levels of unhealthy behaviors. The study highlighted several programs for youth affected by parental incarceration that have been successful in alleviating negative outcomes. Researchers also found that poor behaviors continue even after the parent is released, emphasizing the focus on the parent's reentry back into the home.

Negash et al. (2022) examined the challenges families, particularly those from minoritized and stigmatized groups, face in accessing family therapy after incarceration. Drawing on research and clinical experience, the authors present a conceptual framework addressing barriers from both client and therapist perspectives. Key obstacles include stigma around incarceration and mental health, limited therapist training on carceral systems and racial disparities, and structural issues such as transportation, financial strain, and travel restrictions. These barriers contribute to limited engagement in therapy. To address them, authors advocated for community‐based outreach, flexible service models like telehealth, and culturally responsive, strengths‐based approaches. They also recommended reframing therapy as “transition services” to improve engagement. The article stresses the lack of research on family therapy for formerly incarcerated individuals and offers clinical considerations for supporting this population.

The studies in this section broaden the analysis from the formerly incarcerated individual to encompass the relational systems affected by incarceration, particularly children and families. Nguyen et al. (2023) and Hiolski et al. (2019) both show, using ecological frameworks, that the effects of parental incarceration on children are not temporary or fixed by release, that poor health and unsustainable behavioral problems continue even after a parent comes home, showing that reentry is a family‐level process that needs family‐level responses. Negash et al. (2022) elaborate on this understanding within clinical practice by pinpointing the precise obstacles that hinder families, especially those from marginalized and stigmatized communities. In combination with these findings, the structural obstacles they name are not unique to the post‐release community setting but are amplified by every transition point in the incarceration–reentry continuum. When read alongside the Preparation and Transitional studies, Negash et al. make clear that the absence of relational therapy in this population is not simply a gap in research but a product of compounding access barriers that have never been systematically addressed (2022).

### 5.5 Toward a Relational Framework

Although the relational aspects of incarceration are well‐documented, evidence‐based relational and family therapy interventions are predominantly absent from the reentry literature. First, ecological and systems‐level frameworks, such as Bronfenbrenner's ecological systems theory, social support theory, and family resilience frameworks, consistently emerge across studies at all stages, indicating a collective theoretical acknowledgment that incarceration and reentry are relational phenomena. Nonetheless, this theoretical agreement has yet to yield a corresponding array of clinical interventions. Next, the location and timing of intervention consistently influence therapeutic possibilities. Interventions conducted within carceral environments (Armstrong et al. 2018; Brubacher et al. 2023) are inherently preparatory and structurally limited, whereas transitional programs that extend across the release boundary (Menting et al. 2024; Pettus‐Davis 2021) show promise but remain pilot‐level and non‐relational in their therapeutic modality. Post‐release, community‐based studies offer the greatest opportunity for relational depth, yet the absence of tested family therapy models in this context is striking. In addition, race and structural inequality are not peripheral concerns but organizing features of the reentry experience, particularly for people of color and women, whose relational lives are shaped by surveillance, stigma, and systemic disinvestment in ways that conventional therapeutic models rarely address. Finally, the barrier literature (Negash et al. 2022) makes clear that access to any therapeutic service, including relational, is far from assured for this population. A contextual family therapy framework, which explicitly attends to intergenerational relational ethics, the impact of systemic injustice on families, and the importance of culturally responsive care, is well‐positioned to address these layered needs.

## 6 Discussion

This paper explored how relational therapy is discussed in the literature for post‐incarceration. As discussed in several articles (Brubacher et al. 2023; Armstrong et al. 2018; Menting et al. 2024; Pettus‐Davis 2021; Wilson et al. 2024), re‐entry models may take various forms but will often begin treatment in the prison setting and then continue to provide support for a period after an individual's release (Haas et al. 2022). While parent–child family therapy was the main focus for several articles, relational therapy extends beyond parenting and family reunification and includes other relational units such as intimate relationships and intergenerational systems. The evidence gathered in this review underscored the critical importance of receiving support following incarceration to promote successful reentry. Our review examined how relational therapy is represented in the literature on post‐incarceration and reintegration. Because of this, only two studies (Brubacher et al. 2023; Armstrong et al. 2018) explored a theoretical intervention program that was implemented during incarceration that could aid the reentry process. In the following paragraphs, we identify various cross‐study patterns.

One of the recurring themes in the literature is the crucial part that friends and family play in offering post‐release emotional, material, and logistical support (Pettus‐Davis et al. 2017; Valera and Boyas 2019; Wallace et al. 2016). Better mental health outcomes and lower recidivism rates are often best predicted by this support. Despite the consistent emphasis on familial and social support, only a few studies examined relational or systemic interventions, and the primary focus was parent–child relationships and family reunification (Armstrong et al. 2018; Cooper‐Sadlo et al. 2019; Menting et al. 2024). Although these interventions are crucial for supporting children and addressing the intergenerational effects of incarceration, they do not fully include the relational needs of formerly incarcerated individuals.

Moreover, not a single article investigated couple's therapy or dyadic interventions for romantic partners impacted by incarceration. The lack of this research is significant as relational therapy extends beyond the boundaries of family therapy, offering models that address issues of intimacy, emotional regulation, attachment, and trauma, which are the consequences of incarceration. In couple and family therapy, delinquency, crime, and recidivism are conceptualized in relation to multiple systemic factors, including family dynamics, parenting, relational conflict, peer influence, and neighborhood characteristics (Thompson and Datchi 2019). Given the evidence in other domains of relational therapy's efficacy in strengthening interpersonal resilience (Hammer 2012), this represents a critical gap in the field.

### 6.1 Literature on Relational Interventions

Among the limited studies that addressed relational therapy post‐release, Negash et al. (2022) is the only article that appeared in our search within this particular scoping review to explore relational therapy from a clinical and systemic perspective. Their conceptual framework identifies key barriers to accessing therapy, including stigma, therapist inexperience with carceral systems, logistical challenges, and the cultural mismatch between available services and clients' lived experiences. Negash and colleagues advocate for systemic therapy models grounded in a culturally responsive, trauma‐informed framework that emphasizes flexibility which includes telehealth, community partnerships, and rebranding therapy as “transition services.” Furthermore, most interventions remain conceptual or pilot‐stage despite promising examples like Support4Families (Pettus‐Davis 2021), which is guided by the Main Effect Model of Social Support and Family Resilience Framework. There is a lack of empirically tested models like Emotionally‐Focused Therapy (EFT) and Structural Family Therapy (SFT) in the context of reentry. Integrating such evidence‐based theories might address trust issues, trauma‐related attachment, and role definition among couples.

### 6.2 Clinical Recommendations for Marriage and Family Therapists

Relational therapy has the potential to strengthen not only intimate partnerships but also broader family and community relationships (Tadros et al. 2019). Negash et al. (2026) argue that family therapy is underutilized yet very helpful for individuals and families navigating post‐incarceration reentry, highlighting how relational disruptions, stigma, and systemic barriers limit access to care. The authors outline clinical pathways for therapists, including culturally responsive practice, community collaboration, and adaptations to address attitudinal, relational, and logistical barriers, to make systemic therapy more accessible and effective for families post‐release. Similarly, other recent articles stressed the importance of strengths‐oriented, evidence‐based interventions that support the whole family system (Muentner and Charles 2023).

Although individual‐level interventions, including job support, mental health services, and addiction treatment, are often the focus of the literature, this review emphasizes that relational‐level supports are just as critical in maintaining favorable reentry outcomes. For instance, Valera and Boyas (2019) and Meza et al. (2022) demonstrate how strained social connections and familial stress undermine psychosocial well‐being following release, suggesting that successful reintegration cannot be understood apart from relational functioning. Research examining justice‐involved families further shows that incarceration disrupts attachment bonds, coparenting alliances, and communication processes across relational systems (Tadros et al. 2022; Tadros et al. 2021). These findings suggest that reentry should be conceptualized clinically as a systemic transition rather than solely an individual adjustment process.

The stigma surrounding mental illness discourages many from seeking care. Integrating mental health services with general healthcare can reduce this stigma (Boppre and Reed 2021; Tadros et al. 2023). When designing these services, it is essential to consider the stigma perceived by both staff and peers to ensure individuals can safely access mental health support. For many individuals, incarceration is their first experience receiving mental health services. Despite the prison environment being less than ideal for therapy, it is crucial to provide mental health support during this time to ensure they can maintain connections to these services in the community after release, which is vital for successful reintegration.

Programs designed with multilevel intervention strategies, from trauma‐informed parenting classes (Armstrong et al. 2018) to whole‐systems approaches focused on communication and trust‐building (Brubacher et al. 2023), offer flexible models operating across micro (individual), meso (family), and macro (community) levels. Research with incarcerated couples and coparents demonstrates that relational functioning, communication quality, and perceived coparenting consensus are associated with improved mental and physical health outcomes across partners, underscoring the interdependent nature of reentry adjustment (Tadros et al. 2022). Scholarship further highlights how structured relational interventions, including communication skill‐building and collaborative meaning‐making, function as protective factors among couples navigating incarceration‐related stressors (Durante et al. 2023).

We, as authors of this review, want to be clear that some of these recommendations discussed below were derived from the reviewed literature, and some are our recommendations as scholars conducting this review. Thus, we wrote this section as an amalgamation of the articles and our own ideas, based on scholarly works, to provide recommendations for practicing MFTs. Accordingly, MFTs may expand assessment beyond symptom‐focused intake to evaluate incarceration‐related relational disruptions, including attachment injuries, role disorganization, disrupted coparenting dynamics, and experiences of ambiguous loss during separation. Incarceration‐informed genograms or relational timelines can help MFTs map periods of separation, caregiving shifts, and reunification stressors, allowing treatment goals to emerge from systemic interactional patterns rather than individual pathology. Additionally, having formalized internship programs or practicum placements within correctional mental health systems can help MFT students earn contact hours for licensure while providing family therapy in correctional institutions, creating a viable solution that can be put into place with little or no extra cost to mental health budgets (Garofalo 2020).

Intervention adaptation is also warranted given the limited application of established relational models within reentry contexts. Existing evidence‐based approaches may be meaningfully modified to address incarceration‐related relational trauma. For example, Emotionally Focused Therapy interventions may target attachment injuries associated with prolonged separation and disrupted trust, as demonstrated in clinical work applying EFT principles with justice‐involved couples navigating relational betrayal and reconnection (Zhao and Tadros 2025). Structural Family Therapy techniques may assist families in renegotiating boundaries, hierarchies, and caregiving roles during reunification, as illustrated in culturally informed clinical applications with incarcerated families (Tadros et al. 2024). Additionally, the Tadros Theory of Change provides a systemic framework for working with incarcerated populations by emphasizing relational accountability, contextualized meaning‐making, and collaborative restructuring of family roles (Tadros 2019, 2022). Trauma‐informed relational approaches can further help partners reinterpret withdrawal, emotional numbing, or hypervigilance as adaptive responses to carceral environments rather than interpersonal rejection. Therapists may also increase accessibility through relational telemental health services, which have been shown to expand engagement and continuity of care for incarcerated individuals and their families facing structural barriers to treatment access (Tadros et al. 2021).

Collectively, these implications move systemic therapy from a theoretical framework to a practical approach by operationalizing how therapists can assess relational disruption, structure treatment goals around interpersonal resilience, and adapt established systemic interventions to the relational realities of post‐incarceration life. Translating these findings into practice suggests that treatment planning should explicitly position relationships as protective factors. MFTs may incorporate partners or family members earlier in treatment when clinically appropriate, normalize relational strain as an expected consequence of incarceration, and include psychoeducation addressing how institutionalization, surveillance conditions, and survival‐based coping strategies influence emotional expression and intimacy post‐release (Comfort 2008).

### 6.3 Limitations and Future Directions

While this scoping review has several strengths, several limitations must also be acknowledged. The review was restricted to peer‐reviewed, English‐language articles published between 2014 and 2024. This restriction likely excluded valuable community‐based studies, unpublished program evaluations, and non‐English research, contributing to both publication and language bias. Although selectivity is expected in conducting a review like this (Pham et al. 2014), especially a scoping review, a major limitation was that we only utilized the PsycINFO database; therefore, we recommend that future researchers use other databases as well. The use of a single database may have influenced the body of literature identified. A key limitation stemmed from our initial search terms. Using keywords such as “Family therap* OR couple therap* OR marriage therap*” yielded fewer than 10 results, prompting a broader search strategy. As a result, many of the included studies did not prioritize systemic clinical frameworks. Despite using multiple databases and inclusive strategies, relevant studies may still have been missed due to indexing limitations or access issues.

Our inclusion criteria required explicit mention of relational or systemic approaches, which may have excluded theoretically relevant studies framed differently. Additionally, the studies varied widely in design, sample characteristics, measures, and outcomes, complicating synthesis. Most studies were cross‐sectional, limiting our ability to assess long‐term relational or therapeutic outcomes. There was also a disproportionate focus on women, parenting, and family reunification, with limited attention to non‐parenting individuals, romantic partners, and LGBTQ+ relationships. Methodological inconsistencies across studies may also limit the consistency or comparison of findings.

As there are only a limited number of empirically evaluated interventions in the reviewed literature, future research may benefit from expanding its scope beyond parenting‐focused interventions to include relational therapies addressing romantic partners, chosen families, and LGBTQ+ experiences. Longitudinal studies are particularly needed to evaluate the long‐term effectiveness of relational interventions on psychosocial outcomes, recidivism, and relational stability.

Few studies proposed systemic clinical interventions, and even fewer examined systemic clinical models in depth. Six articles in the review discussed or proposed a clinical intervention (Brubacher et al. 2023; Menting et al. 2024; Negash et al. 2022; Pettus‐Davis 2021; Tadros et al. 2022; Wilson et al. 2024). Among these, only one study described the application of a relational therapeutic model, specifically narrative therapy (Tadros et al. 2022). While the reviewed articles did not examine these approaches, future research may assess other models in conjunction with this population post‐ release, such as Solution‐Focused Brief Therapy (Tadros et al. 2025), Emotionally Focused Therapy (Zhao et al. 2025), Experiential Therapy, and Internal Family Systems (IFS).

Programs like Support4Families (Pettus‐Davis 2021), the Better Start program (Menting et al. 2024), and Passport to Freedom (Wilson et al. 2024) focused on supporting currently incarcerated individuals preparing for reentry. However, some studies suggest that individuals may seek therapeutic support only after release, when they face unique, unexpected reentry challenges (Crain et al. 2024; Gurusami 2019). Several studies emphasized the importance of strong post‐release support systems (Maschi and Koskinen 2015; Valera and Boyas 2019; Wallace et al. 2016). Future research may therefore examine how relational or therapeutic models might be adapted to help formerly incarcerated individuals process their incarceration and navigate reentry.

## Data Availability

Data sharing is not applicable to this article as no datasets were generated or analyzed during the current study.
